# Role of aerobic exercise in ameliorating NASH: Insights into the hepatic thyroid hormone signaling and circulating thyroid hormones

**DOI:** 10.3389/fendo.2022.1075986

**Published:** 2022-12-20

**Authors:** Qiuhong Liu, Han Li, Weiwei He, Qing Zhao, Caoxin Huang, Qingxuan Wang, Zeyu Zheng, Xiaofang Zhang, Xiulin Shi, Xuejun Li

**Affiliations:** ^1^ School of Medicine, Xiamen University, Xiamen, China; ^2^ Department of Endocrinology and Diabetes, Xiamen Diabetes Institute, Fujian Key Laboratory of Translational Research for Diabetes, The First Affiliated Hospital of Xiamen University, School of Medicine, Xiamen University, Xiamen, China

**Keywords:** aerobic exercise, deiodinase type 1, non-alcoholic steatohepatitis (NASH), thyroid hormones, bioinformatics

## Abstract

**Aim:**

Triiodothyronine (T3) administration significantly eliminates hepatic steatosis and also has a therapeutic effect on non-alcoholic steatohepatitis (NASH). However, the potential mechanism by which T3-mediated exercise improves NASH is unknow. This study aimed to explore the effect of aerobic exercise on liver injury in NASH.

**Methods:**

Aerobic exercise was conducted to explore the effects of exercise on liver injury in NASH model induced by Atherosclerotic (Ath) diet. Biochemical evaluations, histological staining and real-time PCR were first applied to confirm the amelioration effects of exercise on NASH. RNA-sequencing (RNA-seq) analysis for livers of each group were further used to identify the underlying mechanisms of aerobic exercise. Bioinformatics methods were used to explore the key functional pathways involved in the improvement of liver tissue in NASH mice by aerobic exercise.

**Results:**

Aerobic exercise improved hepatic steatosis, lobular inflammation and fibrosis in NASH mice. multiple inflammation-related pathways were significantly enriched in the liver of NASH group and improved by aerobic exercise. The results of gene set variation analysis (GSVA) showed a higher enrichment score of T3 response signature in NASH mice with exercise. Increased Dio1 expression in the liver of NASH with exercise mice and increased circulating FT3 and FT4 levels upon aerobic exercise were confirmed.

**Conclusions:**

We found that aerobic exercise could significantly reduce hepatic lipid accumulation, inflammatory infiltration and fibrosis progression in the liver of NASH mice. Hepatic thyroid hormone signaling activation and circulating thyroid hormones is potentially involved in the amelioration effect of aerobatic exercise on NASH progression.

## 1 Introduction

Non-alcoholic fatty liver disease (NAFLD) is currently the most common liver disease in the world, with an overall global prevalence of approximately 25%, representing a major global public health challenge and a significant economic burden (1). NAFLD is prevalent on all continents, with the highest reported prevalence in South America (31%) and the Middle East (32%), followed by Asia (27%), with a prevalence of NAFLD in the Chinese of 29.2% (2, 3). NAFLD is an umbrella term for a group of liver diseases, including non-alcoholic fatty liver (NAFL), non-alcoholic steatohepatitis (NASH) with or without liver fibrosis (4). NASH is an advanced stage of NAFLD, on the basis of steatosis, there are also hepatocellular ballooning and lobular inflammation, so it is more likely to progress to cirrhosis and hepatocellular carcinoma (1). Obesity, insulin resistance and many other factors increase fatty acid transport to the liver. Excess fatty acids promote lipotropic, further leading to Endoplasmic reticulum stress, oxidative stress, and inflammation activation (5–7). The hepatic inflammatory response promotes liver fibrosis, which is a major determinant of increased mortality in NASH patients (8).

So far, several drugs have been developed and tested in clinical trials, but there is still no FDA (the US Food and Drug Administration)-approved therapies for NASH patients. The accepted first line of treatment remains the healthy diet and regular exercise (9). The importance of exercise training is further supported by the findings that aerobic exercise is beneficial in improving liver lipid composition and reducing liver fat content in patients with NAFLD (10, 11). In addition, a prospective cohort study by our group demonstrated that regular moderate-intensity exercise significantly reduced intrahepatic triglyceride levels and improved NAFLD by an aerobic exercise of varying intensity in 220 patients with NAFLD (12). These evidences above confirm the beneficial effects of exercise on NASH, but the exact mechanism by which exercise improves NASH largely remains unclear and needs to be investigated in depth.

It is well known that thyroid hormones (THs) are intimately associated with the liver and that hypothyroidism is significantly dose-dependent with the risk of NAFLD (8, 13, 14). A growing number of studies have examined the relationship between THs and liver steatosis, inflammation and fibrosis (15, 16). However, whether THs is involved in the exercise process to improve liver injury in NAFLD patients is still unclear.

In the present study, we investigated the effect of aerobic exercise on liver injury in NASH mice including inflammation, lipid storage and fibrosis, and compared the thyroid hormone signaling between NASH mice and NASH mice with exercise group based on the animal model and bioinformatics analysis. This study is expected to evaluate the beneficial effects of aerobic exercise in ameliorating NASH pathologies and identify thyroid hormone signaling as one of the underlying mechanisms.

## 2 Methods

### 2.1 Experimental animals

C57BL/6 mice were obtained from Shanghai SLAC Laboratory Animal Co (Shanghai, China). The mice were maintained under controlled temperature (21°C–23°C), humidity (55%–60%) and lighting (12 hours light/dark cycles) and given water freedom. Mice were randomly grouped in three groups (n=8/groups) (1): normal chow diet (NCD) control (2); Atherosclerotic diet (Ath) and kept sedentary (3); Ath diet and received aerobic exercise training on a treadmill. In this study, NCD contained 70% carbohydrate, 10% fat and 20% protein. The Ath diet contained 1.25% cholesterol and 0.5% cholate (17). Body weight was measured every 2 weeks. After the exercise training completed, the mice were fasted overnight and sacrificed with sodium pentobarbital anesthesia, liver and blood samples were collected quickly. Livers were weighed and serum samples were kept at −80 °C for further analysis.

### 2.2 Aerobic exercise training

Before the start of exercise training, the mice were first acclimated to the treadmill for 3 days, 30 minutes per day (12 m/min). After acclimatization to the treadmill according to the above protocol, the mice began 8 weeks of running training (12 m/min, 1 hour per day, 5 days per week). Except for the exercise training group, the remaining two groups remained sedentary in their cages. Mice attempting to rest were encouraged to move by gently tapping on their tail and their back.

### 2.3 Biochemical analysis

Serum aspartate amino transferase (AST) was measured with Biochemical Assay Kit (Mindray, Cat.105-000442-00/105-000443-00), according to the manufacturer’s instruction. Blood total cholesterol (TC) and low-density lipoprotein-cholesterol (LDL-C) levels were measured with Biochemical analyzer (Mindray BS240) according to the manufacturer’s instruction.

### 2.4 Real-time quantitative PCR

Total RNA from liver was extracted using the RNA sample Total RNA Kit (Tiangen, Beijing, China). cDNA was synthesis using reverse transcription kit (FastQuant RT Kit, Tiangen) according to manufacturer’s instructions. Real-time PCR was performed using the Roche Light-Cycler 480 with SYBR Premix Ex Taq II (Takara, Dalian, China). Relative expression of the target genes was calculated using the 2−∆∆Ct method (18). Statistical analysis was performed using GraphPad Prism 8.00

### 2.5 Histological staining and scoring

Liver tissue was fixed in 4% paraformaldehyde neutral solution for 24 hours, the liver tissues were divided into two parts, one part was paraffin embedded, 4 um thick sections were cut and stained with hematoxylin-eosin or Masson staining to assess histological features. For Oil Red O staining, frozen liver sections (5μm) were fixed with fixative for 15 minutes and then stained with freshly prepared Oil Red O solution for 15 minutes. The sections were subsequently rinsed again with 60% isopropanol and finally the nuclei were stained with hematoxylin. The staining was examined under the microscope. For immunohistochemistry, paraffin sections (4um) of liver were immunostained with anti-Dio1 (1:200, 11790-1-AP, Proteintech) using a DAB substrate kit (MXB Biotechnologies, Fuzhou, China) and restained with hematoxylin. Images were captured with a Motic VM1 microscope (McAudi, Hong Kong, China). Digital images were processed with Adobe Photoshop (Adobe, San Jose, CA, USA) and quantitatively analyzed with Image J software (National Institutes of Health, Bethesda, MD, USA).

The histological features of the liver were scored using the NAFLD activity scoring (NAS) system (19) based on HE staining. NAS consists of three aspects: steatosis (0-3), lobular inflammation (0-3), hepatocellular enlargement (0-2).

### 2.6 Transcriptome sequencing and bioinformatics analysis of mice liver tissues

To explore the mechanisms of aerobic exercise on the liver of NASH mice, we performed RNA-sequencing (RNA-seq) analysis of mice liver tissue from each group using the “DESeq2” package of R software, genes with adjusted P-values less than 0.05 and absolute values of log2 fold change (log2FC) greater than 1 were considered to be statistically significant. In order to reveal the possible functions of these key genes, we performed GO enrichment analysis through the Database for Annotation, Visualization, and Integrated Discovery (DAVID) (20, 21). Gene set enrichment analysis (GSEA) is an effective and widely used bioinformatics method for detecting consistent changes in the genome between two phenotypes in transcriptome research (22). Therefore, we used GSEA to explore the key functional pathways involved in the improvement of liver injury in NASH mice by exercise.

### 2.7 Liver transcriptome datasets of mice and robust rank aggregation analysis

To explore the abnormal alterations in liver tissue induced by T3, we searched the Gene Expression Omnibus (GEO) database to identify the transcriptomic dataset of liver from mice treated with T3. The datasets we included had to meet the following criteria: 1) transcriptomic datasets from mice liver; 2) identification of more than 30 differentially expressed genes (DEGs) that differed between the two groups; 3) Genome-wide expression profiles must be available in GEO; 4) datasets must contain both T3 treated and untreated mice. In order to efficiently integrate multiple transcriptomic datasets, a robust rank aggregation (RRA) analysis method was used (23). The R package “limma” was used to analyze the microarray data, while the DEGs in the RNA-seq datasets were examined using the “DESeq2” package of R. Genes with absolute values of log2FC>1 and adjusted P-values<0.05 were considered to be statistically significant in the RRA analysis. Since Gene set variation analysis (GSVA) can assess functional pathway or genomic changes at the genome-wide transcriptome level, we assessed the enrichment score of the key transcriptomic signature in the liver of each group of mice by GSVA (24).

### 2.8 Determination of serum free triiodothyronine and free thyroxine after exercise training

To explore the effect of exercise training on serum free triiodothyronine (FT3) and free thyroxine (FT4). C57BL/6 mice were obtained from Xiamen University Laboratory Animal Centre (Xiamen, China). Eight-weeks-old mice were hosted in polyethylene cages and under controlled conditions of the light–darkness cycle (12/12 h). After mice was adapted to the treadmill, Blood was collected after exercise training and the serum was separated at 4°C and 3000g for 20min for the further analysis. The serum was assayed for FT3 and FT4 using the ADVIA Centaur fully automated immunoassay system.

### 2.9 Statistical analysis

Statistical analyses were performed with GraphPad Prism V.8.0 software. All results were presented as the mean ± SEM. Data were analyzed for statistical significance using a two-tailed unpaired Student’s t test. The p value <0.05 was considered statistically significant.

## 3 Results

### 3.1 Aerobic exercise training improves Ath diet-induced liver injury

**Figure 1:**
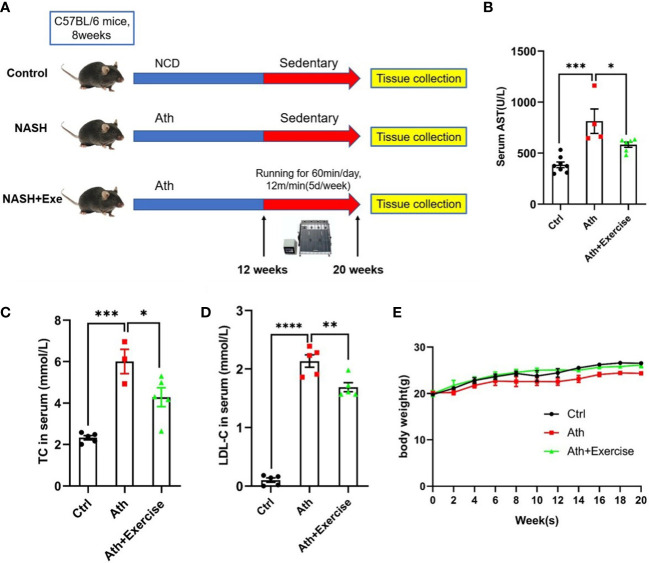
Body weight、liver enzymes and serum lipid metabolism index in mice. **(A)** Schematic diagram of mice running training. C57BL/6 male mice fed an Ath Diet or Normal Chow Diet (NCD) for 12 weeks and then started running training or sedentary training from 20 weeks of age for a period of 8 weeks. **(B–D)** Levels of serum AST **(B)**, serum TC **(C)** and LDL-C **(D)** after 8 weeks of running training. **(E)** Body weight time course. Data are depicted in mean ± SEM. *P<0.05; **P<0.01; ***P<0.001; ****P<0.0001.

To assess the effect of aerobic exercise training on liver injury, we established a NASH model with the Ath diet followed by 8 weeks of treadmill exercise training (**Figure 1A**). Blood sample were collected from mice at the end of the last exercise training for biochemical testing. As expected, we found that Ath diet significantly increased the levels of serum AST, TC and LDL-C in mice. 8 weeks of treadmill exercise training could drastically reduce serum AST, TC and LDL-C levels in NASH mice (**Figures 1B–D**). In addition, we monitored the body weight of the mice throughout and found no significant difference between the groups (**Figure 1E**). The above data suggested that the Ath diet can induce liver injury in mice and aerobic exercise can ameliorate the liver injury of NASH mice.

**Figure 2:**
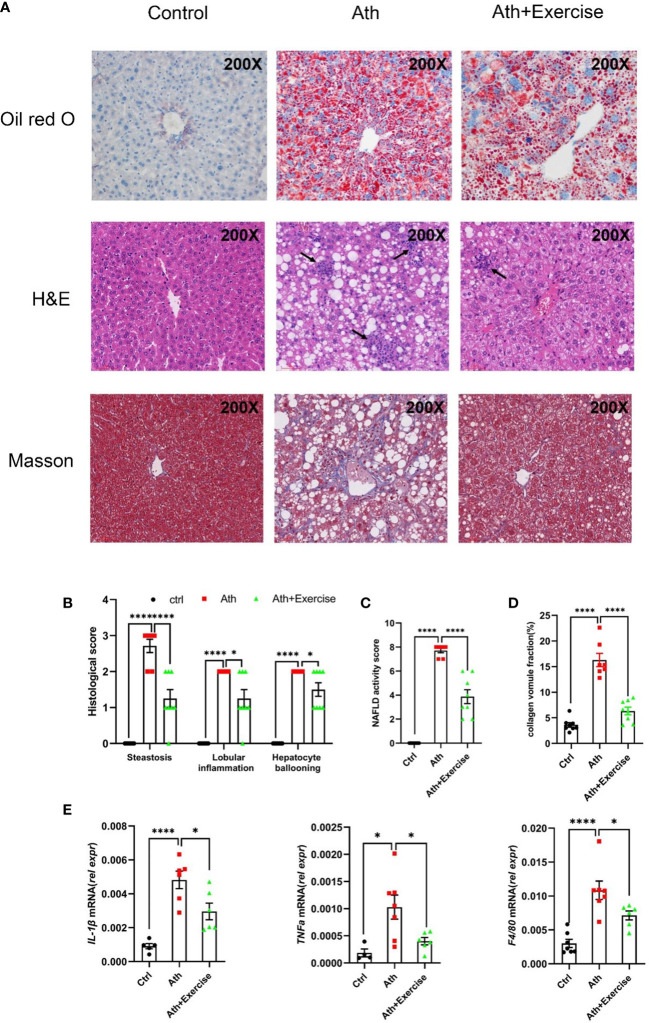
Aerobic exercise decreases hepatosteatosis, lobular inflammation and fibrosis in mouse livers. **(A)** Representative images of liver sections stained by Oil Red O, HE and Masson staining from control, NASH and NASH+Exercise mice (original magnification, ×200). **(B)** Histological scores of steatoses, lobular inflammation, and hepatocyte ballooning in H&E-stained livers. **(C)** NAFLD activity score was calculated by the histological score of steatosis, lobular inflammation, and hepatocyte ballooning. **(D)** The collagen volume fraction by Masson staining. **(E)** Relative mRNA levels of IL-1β, TNFα and F4/80 in liver tissues from control, NASH and NASH+Exercise mice. Data are depicted in mean ± SEM. *P<0.05; ****P<0.0001.

To further evaluate the effect of aerobic exercise training on the hepatic injuries in NASH mice, we measured hepatic histological changes and inflammatory cells accumulation by HE staining, hepatic lipid droplet formation by Oil-Red O staining and fibrosis by Masson staining. In our animal models, severe hepatic lipid accumulation, inflammatory cells infiltration and fibrosis were induced in mice with Ath diet. After 8 weeks of treadmill training, reduced area of hepatic Oil-Red O staining, inflammatory cells infiltration and fibrosis were observed (**Figure 2A**). NAFLD activity score was further determined according to the quantitation of steatosis, lobular inflammation, and hepatocyte ballooning by HE staining. Compared to NCD, large amounts of lipid droplets were observed in the NASH group, and exercise training showed a significant amelioration of steatosis in the liver. Lobular inflammation and hepatocyte ballooning were both obviously visible in Ath-induced mice and were partially improved by aerobic exercise training (**Figures 2B, C**). In addition, collagen fibers were significantly deposited in the liver of mice fed Ath diet, largely eliminated by exercise (**Figure 2D**).

Consistent with the pathological changes shown above, the mRNA levels of inflammation-related genes (IL-1β, TNFα, F4/80) were significantly up-regulated in the liver of NASH mice and down-regulated in NASH mice after 8 weeks of exercise training (**Figure 2E**). There results suggested that 8-weeks aerobic exercise training efficiently improved the Ath-induced hepatic steatosis, lobular inflammation and inhibited fibrogenesis in mice.

### 3.2 Transcriptomic evidence for the function of aerobic exercise in ameliorating hepatic inflammation, lipid metablism and fibrosis in NASH mice

**Figure 3:**
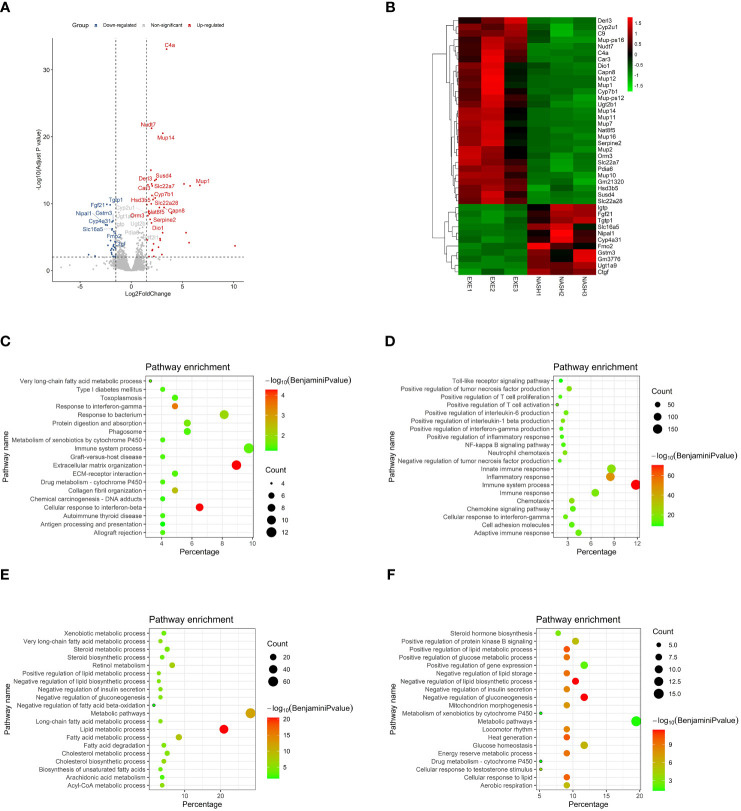
Transcriptomic analyses suggested that aerobic exercise ameliorated hepatic inflammation, lipid abnormity and fibrosis in NASH mice. **(A)** Volcano map of differentially expressed genes in the transcriptomic analyses. **(B)** Heatmap in the transcriptomic analyses comparing NASH mice with NASH+Exercise mice. **(C)** GO enrichment analyses of those crucial down regulated genes in NASH+Exercise mice compared with NASH mice. The significance of enrichment was expressed by the log10 value of Benjamini adjusted P value together with the color transition from white to red. The gene number was for the number of enriched genes in certain functional pathway, while the percentage was for the proportion of enriched genes among total genes in certain functional pathway. **(D)** GO enrichment analyses of those crucial up regulated genes in NASH mice compared with controls. **(E)** GO enrichment analyses of those crucial down regulated genes in NASH mice compared with controls. **(F)** GO enrichment analyses of those crucial up regulated genes in NASH+Exercise mice compared with NASH mice.

To clarify the mechanism of exercise improves injury of liver in NASH mice, we performed RNA sequencing (RNA-seq) on liver tissues of each group and identified a total of 204 DEGs, including 79 genes that were significantly up-regulated in aerobic exercised NASH mice and 125 genes that were significantly down-regulated in aerobic exercised NASH mice. We noted that deiodinase type 1 (Dio1), a key enzyme in thyroid hormone metabolism, was significantly upregulated in aerobic exercised NASH mice (**Figures 3A, B**). GO analyses of the down-regulated DEGs in aerobic exercised NASH mice suggested that these 125 genes were characterized by multiple collagen fibril-related pathways, such as Extracellular matrix organization (Benjamini P=5.34E-5), ECM-receptor interaction (Benjamini P=0.035) and collagen fibril organization (Benjamini P=0.0034) (**Figure 3C**). In addition, Response to interferon-gamma (Benjamini P=2.35E-5) and Immune system process (Benjamini P=0.03) were also enriched (**Figure 3C**). GO analyses of the up-regulated DEGs in aerobic exercised NASH mice suggested that these 79 genes were characterized by multiple lipid metabolic-related pathways, such as positive regulation of lipid metabolic process (Benjamini P=7.40E-11), negative regulation of lipid biosynthetic process (Benjamini P=3.75E-12) and negative regulation of lipid storage (Benjamini P=7.33E-10) (**Figure 3E**). In addition, locomotor rhythm (Benjamini P=5.35E-9), mitochondrion morphogenesis (Benjamini P=3.36E-8) and Aerobic respiration (Benjamini P=2.73E-6) were also enriched (**Figure 3E**).

GO analyses of those up-regulated genes in NASH mice suggested that those genes were characterized by multiple immunity-related pathways (**Figure 3D**), suggesting that advanced inflammatory response in the liver of NASH mice. GO analyses of those down-regulated genes in NASH mice suggested that those genes were characterized by multiple lipid-related pathways (**Figure 3F**), suggesting that lipid abnormity in the liver of NASH mice. The outcomes above suggested that aerobic exercise ameliorated hepatic fibrosis, inflammation response and lipid metabolic abnormity in NASH mice.

**Figure 4:**
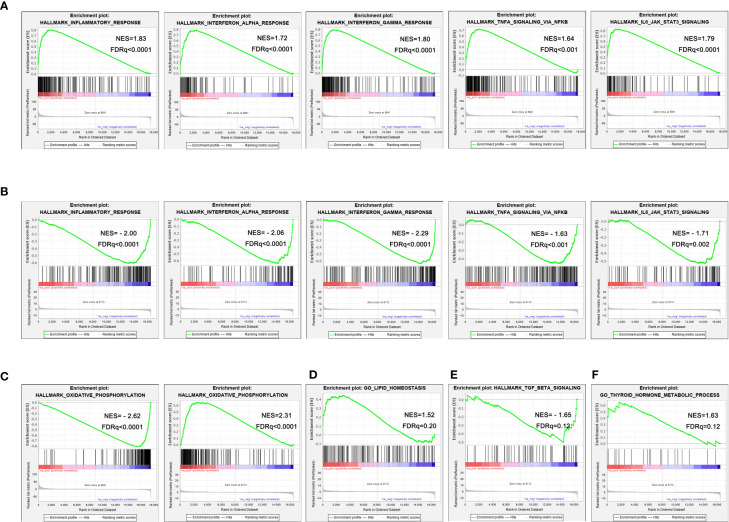
Aerobic exercised NASH mice display improved hepatic inflammation by RNA-seq. **(A)** Representative enrichment plots of important pathways enriched in the liver of NASH mice. **(B)** Representative enrichment plots of important pathways enriched in the liver of NASH+exercise mice. **(C)** Enrichment plots of oxidative phosphorylation signaling pathway enriched in the liver of NASH mice (left) and NASH+exercise mice (right). **(D)** Enrichment plots of lipid homeostasis signaling pathway enriched in the liver of NASH+exercise mice. **(E)** Enrichment plots of fibrosis-related pathway enriched in the liver of NASH+exercise mice. **(F)** Enrichment plots of thyroid hormone metabolic process signaling pathway enriched in the liver of NASH+exercise mice.

The results of GSEA showed that multiple inflammation-related pathways were significantly enriched in the liver of NASH mice, such as Inflammatory response (NES=1.83, FDR q<0.001), Interferon alpha response (NES=1.72, FDR q<0.001), Interferon gamma response (NES=1.80, FDR q<0.001), TNFα signaling *via* NFκB (NES=1.64, FDR q<0.001) and IL6-JAK-STAT3 (NES=1.79, FDR q<0.001) (**Figure 4A**). The enrichment of these inflammation-related pathways was significantly improved in liver tissue from aerobic exercised NASH mice, for example inflammatory responses (NES=-2.00, FDR q<0.001), interferon alpha response (NES=-2.06, FDR q<0.001), interferon gamma response (NES=-2. 29, FDR q<0.001), TNFα signaling *via* NFκB (NES=-1.63, FDR q=0.005) and IL6-JAK-STAT3 (NES=-1.71, FDR q<0.005) (**Figure 4B**). We also found that the Oxidative phosphorylation signaling pathway was remarkably negatively enriched in the liver tissue of NASH mice (NES=-2.62, FDR q<0.001), and oxidative phosphorylation signaling pathway was significantly enriched in aerobic exercised NASH mice (NES=2.31, FDR q<0.001) (**Figure 4C**). In addition, Lipid homeostasis pathway was also enriched in aerobic exercised NASH mice (NES=1.52, FDR q=0.20) (**Figure 4D**). Moreover, TGF_BETA_SIGNALING was significantly downregulated in aerobic exercised NASH mice (NES=-1.65, FDR q<0.02) (**Figure 4E**). Therefore, the above data suggested that there was advanced inflammatory response in the liver of NASH mice, and aerobic exercise can improve inflammation and fibrosis in NASH mice. Notably, we found that Thyroid hormone metabolic process pathway was significantly enriched in the liver of aerobic exercised NASH mice (**Figure 4F**), suggesting that aerobic exercise can enhance thyroid hormone signaling in the liver of NASH mice.

### 3.3 Aerobic exercise increased hepatic thyroid hormone signaling in NASH mice

**Figure 5:**
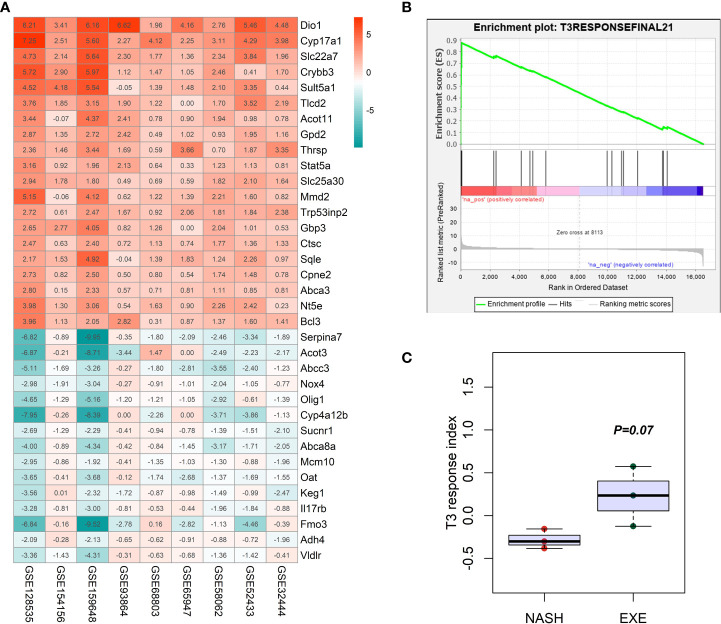
Construction and verification of T3 response signature. **(A)** Heatmap in the RRA analyses of 9 mice liver transcriptome datasets comparing T3 treated mice with non-treated mice. The number was for the log2 value of fold change. Red color indicated the up-regulation of gene expression in the T3 treated mice liver tissues, while green color indicated the down-regulation of gene expression in the T3 treated mice liver tissues. **(B)** GSEA enrichment plot showing the significantly increased enrichment of T3 response signature in the NASH mice liver with aerobic exercise. **(C)** Comparison of the difference in the GSVA enrichment score of T3 response signature between NASH group and NASH+exercise group.

To further explore the role of thyroid hormone signaling pathway in the improvement of hepatic injuries in NASH mice by aerobic exercise, we constructed a T3 response signature to assess the T3 response in the liver of NASH mice. According to pre-defined inclusion criteria in methods, we included a total of 9 mouse liver transcriptome datasets (**Table 1**). These 9 datasets contained a total of 32 T3-treated mice and 32 T3-untreated control mice. We first analyzed individual datasets for differences in gene expression between groups, and then integrated the results of each dataset by RRA analysis, which identified a total of 184 signature genes involved in the T3 response. Of these, 107 genes were significantly up-regulated in the livers of mice treated with T3, notably Dio1 ranked first among the up-regulated genes (**Figure 5A**). We constructed the T3 response signature based on genes which were significantly upregulated in T3-treated mice, including Dio1, Cyp17a1, Slc22a7, Crybb3, Tlcd2, Thrsp, Slc25a30, Mmd2, Sqle, Nt5e, Bcl3, Pnpla3, A1bg, Pnpla5, Fndc5, Ces4a, Adcy10, Sult2a1, Sult1e1, Greb1, Slc13a5.

**Table 1:** Characteristics of mice liver transcriptome datasets in this study.

GSE ID	Samples	Tissues	Methods
**GSE154156**	6 T3 vs 5 Con	Liver	RNA-seq
**GSE159648**	3 T3 vs 3 Con	Liver	RNA-seq
**GSE128535**	4 T3 vs 4 Con	Liver	RNA-seq
**GSE93864**	3 T3 vs 3 Con	Liver	Array
**GSE68803**	3 T3 vs 3 Con	Liver	Array
**GSE65947**	3 T3 vs 3 Con	Liver	Array
**GSE58062**	4 T3 vs 4 Con	Liver	Array
**GSE32444**	3 T3 vs 4 Con	Liver	Array
**GSE52433**	3 T3 vs 3 Con	Liver	Array

RNA-seq, RNA sequencing.

We demonstrated that T3 response signature was significantly enriched in the livers of NASH mice after aerobic exercise by GSEA (NES=1.65, FDR q=0.12), and the results of GSVA showed a higher enrichment score of T3 response signature in the liver of NASH mice after aerobic exercise than NASH mice without exercise (P=0.07) (**Figures 5B, C**). The above results suggested that aerobic exercise could enhance the T3 response in the liver of NASH mice.

### 3.4 Aerobic exercise increased serum FT3 and FT4 levels and hepatic Dio1 expression

**Figure 6:**
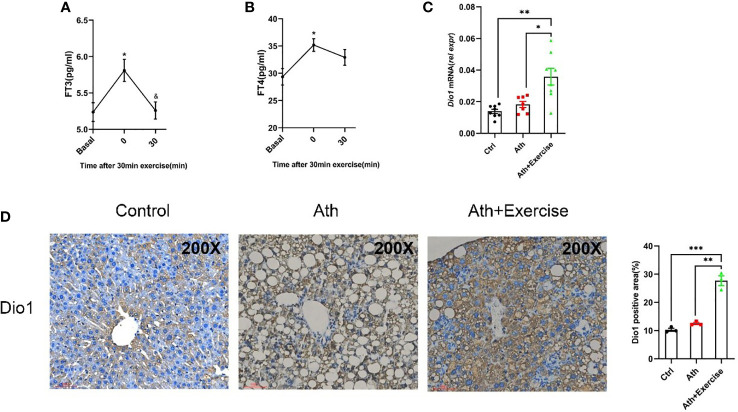
Aerobic exercise increased Dio1 levels in the liver of NASH mice and increased FT3 and FT4 levels in serum. **(A)** The effect of an exercise training on FT3 in mice serum. **(B)** The effect of an exercise training on FT4 in mice serum. **(C)** Aerobic exercise increases the expression of Dio1 in the liver of NASH+exercise mice as verified by q-PCR. **(D)** Immunohistochemistry straining of Dio1 in liver tissues from control, NASH and NASH+Exercise mice (original magnification, ×200). Data are depicted in mean ± SEM. *P<0.05; **P<0.01; ***P<0.001.

In light of the above analysis indicating the involvement of thyroid hormone metabolism mediating the beneficial effect of aerobic exercise on NASH, we further confirm the circulating FT3/FT4 levels in C57BL/6 mice taking treadmill exercise for 30 min. Serum was collected from mice at 0 min and 30 min post-exercise, and the changes of FT3 and FT4 in serum were determined. We noted that there was a significant up-regulation at 0 min post-exercise, followed by a gradual recover to normal levels in 30 min (**Figures 6A, B**). Furthermore, based on the results of the previous RNA-seq analysis, we found that Dio1 expression levels were significantly higher in NASH mice with aerobic exercise training. Considering Dio1 as one of the most key gene in the T3 response signature, we validate hepatic Dio1 expression in the animal models. We examined the mRNA levels of Dio1 in the livers of mice by qPCR and found that Dio1 was increased in the liver of NASH mice after aerobic exercise compared to control mice or NASH mice (**Figure 6C**). We further demonstrated that the positive area of Dio1 positive staining was higher of NASH mice with aerobic exercise by immunohistochemical analysis of paraffin sections of liver tissues (**Figure 6D**). The above results were consistent with the trend of Dio1 expression in the results of RNA-seq. In conclusion, exercise training significantly increased the expression level of Dio1 in the liver of NASH mice after aerobic exercise.

The results suggested that the transient increase of circulating thyroid hormone following exercise may activate the expression of Dio1 in the liver, which may further increase intrahepatic T3 content to form a positive feedback loop.

## 4 Discussion

NAFLD is a hepatic manifestation of the metabolic syndrome. Both obesity and type 2 diabetes rates have continued to increasing prevalence of NAFLD (25). However, there is a subtype of NAFLD, defined as lean NAFLD, which occurs in lean population without obesity with different histological features from those of obese NAFLD (26). A clinical study observed that patients with lean NAFLD had a significantly higher visceral adiposity index than obese NAFLD (27). In this study, we focus on an atherogenic diet containing cholesterol and bile acids. This has been reported to be a more plausible model diet for NASH that significantly induces hepatic steatosis, inflammation and fibrosis (17). In our animal models, Ath diet did not induce weight gain in NASH mice, but caused typical liver pathological changes, including increased liver Oil-Red O staining showing lipid accumulation, and HE and Masson staining showing inflammatory cell infiltration and collagen fibril accumulation. This is consistent with the previous study.

Previous studies have reported that exercise training may improve liver injury in NASH patients, the underlying molecular mechanisms are not fully understood and need to be further investigated (28–30). A study found that the lifestyle intervention group with prominent improvements in liver steatosis, lobular inflammation and hepatocyte ballooning in patients (28). Another study demonstrated that exercise significantly reduced serum ALT levels in NAFLD patients, while improving hepatic steatosis and insulin sensitivity (29). Another randomized trial showed that moderate exercise training distinctively improved hepatic steatosis, lobular inflammation and hepatocyte ballooning by biopsy to detect histopathological changes in the liver (30). A retrospective study demonstrated that moderate to high-intensity exercise improved hepatic steatosis by downregulating the lipid synthesis-related gene Srebp1c and increasing the fatty acid β-oxidation gene CPT1a in peripheral blood mononuclear cells (PBMCs) (31). Moreover, in an animal model of high-fat diet-induced NASH, treadmill exercise significantly reduced hepatic lipid accumulation, reversed liver injury, and inhibited intrahepatic inflammatory responses in mice (32, 33). Therefore, these clinical and animal studies shown above have supported that exercise training is an effective therapeutic strategy for improving NAFLD. Potential molecular mechanisms may be involved including increased hepatic insulin sensitivity, reduced fatty acid synthesis, enhanced fatty acid β-oxidation, reduced hepatocyte apoptosis biomarkers, activation of autophagy and inhibition of excessive production of reactive oxygen species and inflammation-induced oxidative stress (34, 35). In our study, we demonstrated that aerobic exercise significantly reduced the enrichment of inflammatory pathways and fibrotic pathways in NASH mice, and oxidative phosphorylation pathway was significantly enriched in the liver of aerobic exercised NASH mice, which indicated that exercise increased the level of oxidative phosphorylation in the liver.

In this study, an 8-week treadmill training significantly alleviated steatosis, lobular inflammation and improved hepatic injuries in mice. We found that Dio1 was significantly upregulated in the livers of mice in the exercise training group by RNA-seq analysis, which was further confirmed by immunohistochemistry staining and qPCR quantitative analysis. When the body is in motion, muscle contraction affects thyroid function (36). Liver is known to be one of the key target organs for THs, and TH signaling is effective in regulating liver-related disorders, including reducing liver fat accumulation, improving insulin resistance and repairing liver injury (37). T3 levels in the systemic circulation and local tissues are determined by three iodothyronine deiodinases, including DIO1, DIO2 and DIO3. DIO1 and DIO2 convert T4 to biologically active T3, while DIO3 inactivates T4 (38–40). Fortunato RS et al. found a significant increase of T3 levels in rats immediately after 20 min of treadmill exercise, followed by a gradual decrease and a concomitant decrease in Dio1 enzyme activity in the liver (41). Recently, another study found that the expression level of Dio1 increases in the early stages of NASH and regulates hepatic triglyceride levels in the liver (42). However, the role and mechanism of Dio1 in exercise amelioration of NASH is still unclear.

To clarify the effect of exercise on intrahepatic T3 response, we constructed a T3 response signature and found that the T3 response signature was remarkably enriched in the livers of NASH mice after exercise by GSEA and GSVA. The above results highlighted that exercise can significantly enhance the intrahepatic T3 response. There is growing evidence showing that hypothyroidism is associated with an increased risk of NAFLD (43). A multicentre study that included 20 male patients revealed that patients treated with low doses of levothyroxine showed a 12% reduction in intrahepatic lipid content from baseline and a small reduction in visceral adipose tissue volume and subcutaneous adipose tissue volume (44). It is important to note that recent clinical trials have shown that the use of agonists such as Resmetirom (MGL-3196) targeting THRβ can significantly reduce liver fat content (45). In animal model studies, T3 restored liver mitochondrial biogenesis and autophagy levels in NASH mice thereby increasing fatty acid β oxidation and also reducing liver inflammation and fibrosis (16). Since T3 increases the expression level of DIO1 in the liver (46), in order to explore the reason for the increased level of Dio1 in the liver of mice after exercise, animal studies were performed to measure serum FT3/FT4 levels. The results showed that serum FT3/FT4 increased immediately after exercise training. It has also been previously reported in the study that serum levels of T3, T4 and T3/rT3 were increased in rats after treadmill running training (41, 47). Collectively, these findings support the notion that serum thyroid hormone levels can be modulated by exercise.

Dio1 expression and activity are influenced by hormonal and secondary messengers, nutritional and developmental factors and species differences, with THs being the most important impact factor (48). M Menjo et al. demonstrated that T3 can act directly on rat hepatocytes to increase the mRNA expression level of Dio1 (49). In another *in vitro* study, T3 increased the mRNA level of Dio1 by 76 ± 17% and the activity of Dio1 by 101 ± 30% in rat hepatoma cells (50). Similarly, in the streptozotocin (STZ)-induced type 2 diabetes rat model, Dio1 activity and mRNA expression level in rats recoved to control levels after administration of T3 treatment and increased significantly when T3 and insulin were co-administrated (51). Our current study found that serum FT3 and FT4 were elevated immediately after treadmill exercise in mice, while the expression of Dio1 in the liver was also significantly upregulated after aerobic exercise on treadmill, which was consistent with the results of several previous *in vivo* and *in vitro* experiments. The above results suggested that the increase in serum T3 levels after exercise may continuously increase the expression of Dio1 in hepatocytes.

In conclusion, our study provides evidence that exercise training significantly improved hepatic lipid accumulation and also reduced inflammatory infiltration and fibrosis. In addition, we constructed T3 response signature using mice liver transcriptomic datasets and found that aerobic exercise significantly increased T3 response in the liver of NASH mice and Dio1 was the most important molecule in the T3 response signature. We confirmed transient elevation of FT3 after exercise and upregulation of Dio1 expression level in the liver of NASH mice post aerobic exercise, which may partially mediate the protection effect of exercie on hepatic injuries in NASH mice.

## Data availability statement

The data presented in the study are deposited in the GEO dataset repository, accession number (GSE217155).

## Ethics statement

The study was approved by the Ethics Committee of the First Affiliated Hospital of Xiamen University.

## Author contributions

All of the authors contributed to the design of the study. QL and HL wrote the manuscript; XL, and XS designed the study; WH and QZ generated the figures; QW, ZZ, and XZ performed the research; CH reviewed and edited the paper. All the authors agreed to the published version of the manuscript.
