# Corrigendum: Role of aerobic exercise in ameliorating NASH: Insights into the hepatic thyroid hormone signaling and circulating thyroid hormones

**DOI:** 10.3389/fendo.2023.1146843

**Published:** 2023-01-30

**Authors:** Qiuhong Liu, Han Li, Weiwei He, Qing Zhao, Caoxin Huang, Qingxuan Wang, Zeyu Zheng, Xiaofang Zhang, Xiulin Shi, Xuejun Li

**Affiliations:** ^1^ School of Medicine, Xiamen University, Xiamen, China; ^2^ Department of Endocrinology and Diabetes, Xiamen Diabetes Institute, Fujian Key Laboratory of Translational Research for Diabetes, The First Affiliated Hospital of Xiamen University, School of Medicine, Xiamen University, Xiamen, China

**Keywords:** aerobic exercise, deiodinase type 1, non-alcoholic steatohepatitis (NASH), thyroid hormones, bioinformatics

In the published article, there was an error in the Funding statement. The funding reference 2021J011363 should have not been included in the article.

The correct Funding statement appears below.

